# Protective socks for people with diabetes: a systematic review and narrative analysis

**DOI:** 10.1186/s13047-015-0068-7

**Published:** 2015-03-27

**Authors:** Simon J Otter, Keith Rome, Belinda Ihaka, Andrew South, Mandy Smith, Amit Gupta, Frances Joseph, Peter Heslop

**Affiliations:** School of Rehabilitation & Occupation Studies, AUT University, North Shore Campus, Private Bag 92006, Auckland, 1142 New Zealand; Fashion Design, School of Art & Design, AUT University, WW Building, 9 Mount Street, 1010 Private Bag 92006, Auckland, 1142 New Zealand; Textile and Design Laboratory, AUT University, WW Building, 9 Mount Street, 1010 Private Bag 92006, Auckland, 1142 New Zealand

**Keywords:** Diabetes, Diabetic foot, Offloading, Footwear

## Abstract

Padded socks to protect the at-risk diabetic foot have been available for a number of years. However, the evidence base to support their use is not well known. We aimed to undertake a systematic review of padded socks for people with diabetes. Additionally, a narrative analysis of knitted stitch structures, yarn and fibres used together with the proposed benefits fibre properties may add to the sock. Assessment of the methodological quality was undertaken using a quality tool to assess non-randomised trials. From the 81 articles identified only seven met the inclusion criteria. The evidence to support to use of padded socks is limited. There is a suggestion these simple-to-use interventions could be of value, particularly in terms of plantar pressure reduction. However, the range of methods used and limited methodological quality limits direct comparison between studies. The socks were generally of a sophisticated design with complex use of knit patterns and yarn content. This systematic review provides limited support for the use of padded socks in the diabetic population to protect vulnerable feet. More high quality studies are needed; including qualitative components of sock wear and sock design, prospective randomized controlled trials and analysis of the cost-effectiveness of protective socks as a non-surgical intervention.

**Electronic supplementary material:**

The online version of this article (doi:10.1186/s13047-015-0068-7) contains supplementary material, which is available to authorized users.

## Introduction

Diabetes mellitus, particularly the mature onset or type-two variation (T2DM) is a major health concern world-wide [1-3]. T2DM is a cause of significant co-morbidity and is predicted to further increase over the next 20 years contributing to a greater diabetes-related burden [1,4-6]. Some 15%-25% of people with diabetes will suffer a foot ulcer [7,8] and limb amputation is preceded by foot ulceration in 85% of cases [9]. More worryingly it is suggested that some 80% of amputations are preventable [10]. The aetiology of foot ulcers is complex and has been extensively reviewed [11-14]. Complications (including vascular disease, peripheral neuropathy, increased mechanical stress and Charcot neuroarthropathy) greatly increase the incidence of lower limb amputations [14-17]. However, the nature of diabetes means even those at low risk can develop foot complications [18], particularly in the presence of poor glycaemic control and/or a lack of regular foot assessment. Foot complications negatively impact individuals’ quality of life and their ability to be productive members of society and these complex pathologies are a considerable health system burden [19-21].

A range of non-surgical approaches can be used to prevent the foot complications seen in diabetes including education, self-care/self-monitoring of feet, appropriate skin and nail care, wearing supportive footwear and protective socks, as well as formal podiatric assessment and treatment [22-24]. From a commercial perspective a vast range of protective socks are commercially available (Additional file 1). Recent studies in diabetic foot ulcer prevention have reported on foot orthoses and footwear to reduce foot pressure [25,26]. A systematic review [27] considered ‘socks for people with diabetes’ but did not present any formal scoring, yet determined results for this type of hosiery were inconclusive. A recent Cochrane review appraised off-loading strategies, but did not include protective socks [28]. Therefore, we sought to undertake a systematic review of protective socks for people with diabetes and included a narrative analysis of these socks, which included an analysis of the knitted stitch structure and yarn/fibre type.

## Review

### Methods

This systematic review was undertaken according to the guidelines provided by the Cochrane Collaboration [29] and the PRISMA group [30].

#### Search and selection process

To obtain all articles relating to the use of socks for people with diabetes an extensive literature search was designed jointly by the lead author (SO) and an experienced librarian (AS) performed across several databases EBSCO (Biomedical reference collection, Cinhal, Health business elite, Health source, Medline, Sport discuss) SCOPUS, AMED, Cochrane and PEDro. Databases were searched from 1985 – 2014, as socks for people with diabetes were not available prior to 1985. Further searches of manufacturer’s websites were also conducted.

**Figure 1:**
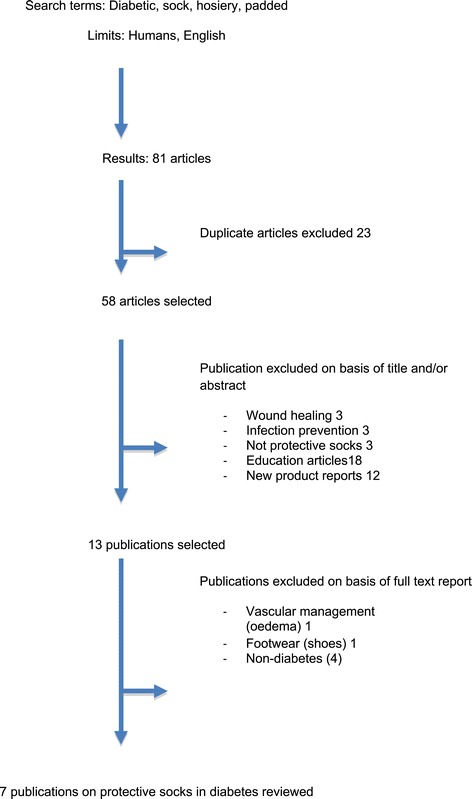
**PRISMA diagram to show selection of publications.**

Publications were identified through a search that used the following MeSh terms: (diabet* AND sock) OR (diabet* AND socks) OR (diabet* AND hosiery) OR (diabet* AND padded). Articles were limited to “humans” and “English”. Inclusion criteria comprised articles reporting any type of clinical trial design, including people with diabetes reporting the use of socks for people with diabetes. Articles were initially excluded if they did not report diabetes; did not focus on socks for people with diabetes as a mechanism for foot protection reduction, (for example articles reporting the use of hosiery to control oedema). Articles relating to non-diabetic groups, for example, healthy populations or sports were also excluded. Owing to a paucity of high quality research, studies were included if they were of level four or above [31] and in English language. Reviews, editorials, letters and single case histories were excluded. The selection process was performed on the titles of articles, the abstract then on full text (Figure 1).

#### General data extraction

Publications were evaluated based on the full text article and reviewers were not blinded to the journal title or authors. Information was extracted based on year of publication, study design and number of participants. Demographic data such as gender, age, duration of diabetes, disease features and length of follow-up was also recorded. Publications were reviewed with the use of a tool developed by Downs & Black [32]. While the Cochrane Collaboration [29] does not recommend a specific tool for non-randomised clinical trials, this instrument has been widely used for non-pharmacological trials and provides a score between 0-32 across 27 questions: a higher score representing robust, high quality methodology. The tool is easy to complete with high internal consistency, test-retest and inter-rater reliability reported to be good by the authors [32]. The checklist covers study quality (10 items), external validity (3 items), bias (7 items), confounding and selection bias (6 items) and the power of the study (1 item) [33].

#### Statistical Analysis

Analysis was mainly descriptive based on an analysis of the narrative provided by studies, i.e. the extent to which plantar pressures were reduced; together with the Downs and Black score for each article. In this review four questions (7, 15, 16 and 25) were omitted from the Downs & Black tool as these were not applicable to any of the trials being reviewed. Each score is therefore expressed as a percentage to permit ease of comparison.

### Description of publications

A total of 81 articles were retrieved, but we identified only seven prospective studies [34-40] that met the inclusion criteria. A further five papers [41-45] considered the role of protective socks in other populations (e.g. athletes’ or rheumatoid arthritis). While these findings may be transferrable to the diabetic population, they were excluded from the review. From the additional 70 articles that were excluded, many were duplicates (n = 23). Others were industry reports highlighting new product developments (n = 12), but not providing any empirical evidence to support the product (Figure 1). A further 18 papers consisted of education and/or continuing professional development articles for health professionals highlighting the need to protect the ‘at risk’ diabetic foot. Of the seven studies included, three were case series, three cross sectional designs and one single blind RCT. The main characteristics of the studies reviewed are presented in Table 1. The mean quality score was 39% (SD 20, range 17-78%) - details in Table 2.

**Table 1:** **Overview of studies reviewed**

**Study**	**Demographic data**	**Inclusion criteria (in addition to diabetes)**	**Findings**
Blackwell et al. [34]	N° of subjects 21	Diabetes with foot complaints, no active ulceration	Plantar pressure assessed with Parotec system
	Gender 10 M : 11 F		
	Mean age (range) 57.4 (20-83)		No significant difference between *JBOST* diabetic sock, normal sock or barefoot
	Diabetes duration Not stated		
Veves et al. [35]	N° of subjects 27	High plantar pressures (>10 kg/cm^2^)	Plantar pressure assessed with optical pedobarograph
	Gender 15 M: 2 F	Neuropathy (diminished nerve conduction & vibration perception)	Experimental socks [*Thorlo*] provided significant pressure reduction compared with pts own socks or barefoot (both p < 0.001)
	Mean age (range) 54 (26-74)		
	Diabetes duration not stated	Able walk unaided, no PVD, no ulcer history	
Veves et al. [36]	Gender not stated	Neuropathy (diminished vibration perception & absent ankle reflex)	Plantar pressure assessed with optical pedobarograph
	Experimental group (n = 10)		
	mean age (range) 51.3 (27-65)		
	Duration of diabetes not stated		Significant reduction in pressure of experimental socks [*Thorlo*] compared with padded sports socks & barefoot (all p < 0.001). Pressure reduction maintained by experimental socks at 3 & 6 months.
	Control group n = 16		
	Mean age (range) 55.8 (33-70)		
Garrow et al. [37]	N° of subjects 19	Neuropathy (neuropathy disability score >5 or diminished vibration perception ≥25).	Plantar pressure assessed with F-scan system
	Gender 15 M:4 F		
	Mean age (range) 65.5 (39-80)	Ulcer-free at recruitment	*Preventative Foot Care* Diabetic socks provided significant increase in foot contact area (p < 0.01), a reduction total pressure (p < 0.01).
	Diabetes duration median 20 yrs		
		High plantar pressure (≥6 kg/cm^2^).	
Murray et al. [38]	N° of subjects 86	Neuropathy (diminished pressure or vibration perception)	Questionnaire based satisfaction survey over 6 month period using *Thorlo* socks
	Gender 69 M :17 F		
	Mean age (range) 63 (34-85)	No active ulceration	Socks reported good/very good by 86%, average by 12% & poor by 3%.
	Mean diabetes (range) 16 (1–45 yrs		84% reported continue sock use at 3 & 6 months
Banchellini et al. [39]	N° of subjects 30	Peripheral neuropathy (ADA criteria)	Skin parameters tested:
	Gender not stated		Hydration (hydration score) Hardness (Durometer)
	Group A (*Difoprev*) socks	Anhidrosis (Clinical features & Neuropad test)	Moisture loss (Scalar moisture checker)
	Mean age 59.6 (SD13.8)		Water loss (TEWL vapometer)
	Duration diabetes 16.1 (SD9)	No active ulceration, ABPI >0.9, Serum creatinine >2 mg/dL	All skin parmeters improved over 6 week trial (Difoprev) socks & normal socks
	Group B (no active sock ingredient)		
	Mean age 61.4 (SD15.5)		Skin hydration p < 0.01 Skin hardness p <0.05
	Duration diabetes 15.7 (SD6.9)	No systemic skin disease, no *B*-blocker therapy	
	Controls (normal socks)		Skin moisture loss p < 0.01 Skin water loss p < 0.01
	Mean age 60.5 (SD11.4)		
Yick et al. [40]	N^o^ of subjects 4	No inclusion criteria stated	Plantar pressure (Pedar system)
	Gender not stated		Skin temperature & humidity (system not stated)
	Age not stated		Socks tested not stated
	Diabetes duration not stated		Considerable pressure reduction stated but not per sock type
			Thermal properties are stated but not compared between socks or post sock wear

**Table 2:** **Description of scoring based on Downs & Black criteria**

**Study**	**Is the aim of the study clear?**	**Are the main outcomes clearly described?**	**Are characteristics of patients included clearly described?**	**Are the interventions clearly described?**	**Are co-founders clearly described?**	**Are the main findings clearly described?**	**Have adverse events been reported?**	**Are subjects lost to follow up characteristics reported?**	**Are actual probability values reported?**	**Were subjects invited representative of population**	**Subjects who participated representative of population?**	**Were staff, places & facilities representative?**	**Was there an attempt to blind study subjects?**	**Were analyses adjusted for different lengths of follow-up between interventions?**	**Were appropriate Statistical tests used?**	**Was compliance with interventions reliable?**	**Were main outcome measures accurate and reliable?**	**Were cases/controls recruited from same population?**	**Were cases/controls recruited over same time?**	**Were subjects randomized to intervention groups?**	**Was randomized intervention concealed form subjects & clinicians?**	**Are analyses adjusted for lost to follow up subjects**	**Study have sufficient power to detect clinically important effect?**	**Total score (%) based on 23 items**
Veves et al. [37]	X	X	X	X	O	O	X	U	O	U	X	X	O	U	X	X	X	U	U	U	U	U	O	10 (43.5%)
Veves et al. [38]	X	X	X	X	O	X	X	U	O	U	U	X	O	O	X	X	X	U	U	U	U	U	O	10 (43.5%)
Murray et al. [40]	X	O	X	X	X	O	X	U	O	U	U	U	O	U	U	X	U	U	U	U	U	U	U	6 (26.1%)
Blackwell et al. [36]	X	O	O	X	O	O	O	O	O	U	U	U	O	U	X	X	X	U	U	U	U	O	O	5 (21.8%)
Garrow et al. [39]	X	X	X	X	X	X	U	O	O	U	U	X	O	U	U	U	X	U	U	X	O	O	U	9 (39.2%)
Banchellini et al. [41]	X	X	X	X	X	X	X	U	O	X	X	X	X	X	X	X	X	X	X	X	O	U	U	18 (78.3%)
Yick et al. [42]	X	X	O	X	O	O	O	U	O	U	U	U	O	O	U	U	X	U	U	U	U	U	O	4 (17.4%)

X = yes, O = no, U = unable to tell.

Questions omitted: 7 - no trials reported the random variability for their main outcomes, 15 - none of the studies were double-blind, 16 - there was no evidence of data dredging, 25 - cofounding variables were not adjusted for throughout.

### Plantar foot pressure

Five studies [34-38,40] used peak plantar pressure as the primary outcome measure. A variety of protective socks were included. Additionally, most studies also included a control element with subjects using their own socks or standard shop-bought socks together with barefoot pressure measurements as a true control condition. Three studies [35-37] reported padded socks provided a significant reduction in peak plantar pressure. They suggest that in conjunction with wearing proper footwear/orthoses, padded socks could help prevent foot ulcer formation. However, one study [34] reported an increase in peak plantar pressure with padded socks. One study reported a follow-up period [36] and a significant reduction in peak plantar pressure was maintained at 6 months, although this reduction was not as great as was seen at baseline.

### Plantar contact area

One study [40] reported plantar contact area as an outcome and reported a significantly greater contact area with socks for people with diabetes compared with ordinary shop-bought socks. These authors demonstrated an increase in maximum foot contact area of 11 cm^2^ when subjects wore the protective socks, accompanied by a 9% reduction in total foot pressure. Similar results were observed at the forefoot, a 14% increase in contact area and 10% reduction in peak forefoot pressure.

### Patient satisfaction

One study used a survey design approach to quantify how satisfied subjects were with socks designed to reduce pressure over a 6-month period [41]. The results were positive; with 85% reporting high satisfaction, and 84% of participants reported they wished to continue wearing the socks after the trial.

### Skin moisture and temperature

Banchellini et al. [39] reported in a 6-week randomised trial into a new nanotechnology impregnated sock design intended to increase skin moisture content. The sock (Difoprev system, LVM technologies Italy) consisted of a synthetic polyammide fibre loaded with microcapsules of an emollient agent. Additionally, Yick et al. [40] noted an increase in skin temperature and humidity with protective socks in a sample of two subjects with diabetes.

From this review there is weak evidence that protective socks may reduce foot pressures and provide additional protection for the at-risk foot in diabetes. There are four domains (plantar pressure, plantar contact area, satisfaction and skin moisture) that reflect relevant clinical outcomes and are reported in research articles over 25 years. However, in spite of sophisticated sock design and material usage employed by manufacturers, studies received low scores using the Downs and Black instrument. The majority of studies compared very small populations and were not adequately powered. This together with limitations in the overall design (e.g. lack of randomization, blinding of participants and/or clinicians) also contributed to low scores. While it is difficult to blind clinicians working in healthcare settings, the guidance offered by Boultron et al. [46] and Cook [47] are essential as a lack of non-blinded assessors can cause a high risk of observer bias [48]. In most studies some attempt was made to identify participants with diabetes who would benefit more from protective socks (i.e. those with higher plantar pressures and loss of protective sensation), which might also suggest greater improvements would be reported. However, not all articles controlled for the complications commonly seen in diabetes (e.g. vascular disease, current foot ulceration or previous amputation). While this may represent the heterogeneous nature of foot complaints seen in diabetes, equally there were no clear attempts to include adequate numbers of subjects with these complications to represent the heterogeneous nature of foot complaints seen in the diabetic population. Moreover, the contention of many articles was that reducing foot pressure would prevent foot ulceration. However, the incidence of foot ulceration was not a primary outcome measure and not always stated as an adverse event.

Plantar pressures have long been recommended as a key outcome measure to identify those at risk of foot ulceration [17,49]. Notably, up to three-quarters of foot ulcers are over the metatarsal head region [50] – often an area of high pressure. Ulbrect et al. [26] report that peak barefoot plantar pressure is the key determinant when manufacturing bespoke orthoses to off-load pressure. A significant reduction in plantar pressure was reported by three studies while using protective socks [35-37]. However, considerable variations between peak plantar pressure values have also been reported for those with and without foot ulcers [26,51,52]. These differences may be due to a number of factors, including the protocol and equipment used. In the articles we reviewed, research protocols were often not clearly described. That said, many were published prior to the development of guidelines for plantar pressure studies [53]. Additionally, footwear is often a key therapeutic intervention; [27,54] so controlling for, or standardising footwear should also be a consideration when designing research protocols when testing protective socks. Foot structure, biomechanics and tissue glycation may have a marked effect on plantar pressure variables [55-57] and these variables should also be considered either as part of the exclusion criteria or as potential confounders when assessing the impact of protective socks on plantar pressures.

Satisfaction and concordance with interventions is a key area for research, as patients are unlikely to continue to wear socks they are unhappy with or find uncomfortable. Only one study [38] addressed this important aspect of practice, but the instrument used to determine satisfaction was not provided, making an adequate assessment of its appropriateness, responsiveness and reliability difficult. Any changes in clinical outcomes (e.g. a reduction in plantar pressure) that may have occurred during the study period was not reported.

From the narrative analysis of articles and website data, all socks reviewed were knitted using a weft knit method with a variety of yarns (Additional file 1). We noted sophisticated sock designs that included the use of pile fabric knit structures over areas requiring extra padding to reduce pressure, rib knit structures used to provide compression and support structure over the ankle and mesh or tuck knit structures allowing for free ventilation where less protection and greater flexibility is needed. Bertaux et al. [58] reported significant correlations between physiological and sensory parameters as well as between fabric friction and perceived comfort in eleven subjects wearing sports socks. This highlights that ‘comfortable’ socks provide lower friction coefficients and hence reduce the potential for skin damage. Maximising protection and reducing friction at the foot/sock interface is thought to be key for preventing lesions in the at-risk diabetic foot [59]. However, parameters such as shear and temperature were typically not comprehensively studied in the articles we reviewed. While not a padded sock *per se*, the Difoprev system reported by Banchelline et al. [39] provided a significant increase in skin hydration. A decrease in moisture loss, water loss and hardness is of value to people with insensate feet, where autonomic neuropathy in particular is known to cause excessive dryness and is a risk factor for foot ulceration [13,60].

This paper represents the first review to combine a systematic review on a topic that has not been previously addressed, together with a narrative analysis of the key intervention. There are some limitations to consider. Ideally a meta-analysis would be conducted in conjunction with this systematic review. However, this was not possible as the studies using plantar pressure analysis (the primary outcome in the majority of studies) were conducted with various systems to measure foot pressure. This results in different spatial and temporal resolutions, data extraction and management approaches. Additionally, the main plantar pressure variable was reported differently throughout. These factors make comparisons between trials difficult and not conducive to further statistical analysis.

## Conclusion

Altering the socks people with diabetes wear could provide a simple, cosmetically acceptable, and potentially cost-effective method of protecting the at-risk foot in diabetes. However, the previous studies of protective socks were often poorly controlled, underpowered and did not justify the primary outcomes reported. Consequently, there are opportunities for further research, including qualitative components of sock wear and sock design, together with randomized controlled trials and analysis of cost-effectiveness.

## Additional file

Additional file 1:
**An overview of commercially available protective socks, their properties, manufacturer’s claims and supporting evidence.**
