# Supplementary material for: Protective socks for people with diabetes: a systematic review and narrative analysis
Source: J Foot Ankle Res. 2015 Mar 27;8:9. doi: 10.1186/s13047-015-0068-7 (PMC4431172; doi:10.1186/s13047-015-0068-7)
Supplement: Additional file 1: — An overview of commercially available protective socks, their properties, manufacturer’s claims and supporting evidence. [file 13047_2015_68_MOESM1_ESM.docx]

| **Sock type/Name** | **Manufacturer** | **Cost** | **Material content** | **Manufacturer’s claims** | **Supporting Evidence** |
| --- | --- | --- | --- | --- | --- |
| Thorlo Padded Socks  **Thorlos MEDDS® (only on prescription for individuals diagnosed with diabetes & at risk of ulceration or amputation)** | Thorlo USA  [www.thorlo.com/activity/therapeutic/padds-socks](http://www.thorlo.com/activity/therapeutic/padds-socks) | Unisex Padds® over-calf socks – USD $19.99  Unisex Padds® crew sock with THOR.LON ® -USD $16.99 | ‘Thorlon’ acrylic yarn  Details not states | Provide foot protection and comfort to people with diabetes whose feet are not at immediate risk of ulceration or amputation  **Shin:** Constructed with THOR•LON® fibers for superior softness, resilience, durability and moisture-wicking for drier, better feeling feet.  **Calf:** Over-the-Calf length has supportive top with tighter elastic to help reduce “tired” feeling feet and legs.  **Ankle**: Crew and min-crew lengths have relaxed fit at top for added stretch and more comfort.  **Heel**: Unique padding specifically designed for everyday activities, clinically shown to reduce blisters, pain, pressures and moisture for maximum protection  **Instep:** Flat knit instep and cushioned arch for a better fit & support for more comfort.  **Toe:** Low profile seam won’t rub or irritate feet | KM Herring & DH Richie (1990) Friction Blisters & Sock fiber composition: double Blind study. JAPMA 80:2: 63-71  KM Herring & DH Richie (1993) Comparision of cotton & acrylic socks using a generic cushion sole design for runners. JAPMA 83:9:515-522  A. Veves, et al (1992) The use of specially padded hosiery in the Painful Rheumatoid foot. The Foot 1 175- 177  A. Veves, et al (1989) Use of Experiemental padded hosiery to reduce abnormal foot pressure in Diabetic Neuropathy diabetes Care 12:9: 643 – 655  A. Veves, et al (1990) Studies of Experimental Hosiery in Diabetic Neuropathic Patients with High Foot Pressures Diabetic Medicine 1990:7:324-326  S. Flot, et al (1995) The Effect of Padded Hosiery in Reducing forefoot Plantar Pressures The Lower Extremity 2:3:201 -205 |
| **TXG Compression Stockings:**  TXG Energy Preventative Compression socks  TXG Diabetic Cushion Socks: | Taiwan  <http://txgsocks.com/en/>  [www.txgsocks.co.nz/product/sensitive-foot/](http://www.txgsocks.co.nz/product/sensitive-foot/) | TXG Compression socks “Sensitive Foot” 15-20mmHg -  NZD $59.00  Diabetic Cushion –  NZD $20.00 | 74.9% Acrylic  15.4% Nylon  7.3% polyester  2.4% Spandex (Lycra®)  84% Acrylic  7.5% Spandex (Lycra)  8.5% Nylon | **TXG Energy Preventative Compression socks:** non-severe diabetic foot, compression socks made from anti-bacterial fiber are helpful in lowering extremity edema, bacterial propagation avoidance and hasten the wounds recovery  **Calf**: wide Stripe design giving no oppressive injuries  **Ankle**: Mesh-like ventilation designed for comfort & superb ventilation  **Toe**: Bamboo charcoal yarn is added to be antibacterial deodorant  Provide compression level 10-15mmHG  **Diabetic Cushion Socks:**  Designed not to stimulate the skin and cause friction wounds, inflammation or ulceration. Antibacterial socks with zero compression are most suitable for severe diabetic patients with diabetic foot problems. Antibacterial acrylic yarn can keep bacteria away. Bacteria cannot spread & infest in artificial materials unlike natural materials like cotton. In the design of the diabetic foot socks, soft contact with fabrics and design of seamless access any possible wounds caused by friction.  **Calf**: Special weave process allows sock leg stretch up to 22cm gives snug fit without oppression on skin  **Instep**: Ventilation design for extra comfort  **Toe & Heal**: Enhanced padding area for complete toe to heal wrap around  Provides compression: 0mmHg |  |
| LifeSock  NZ sock Company | Ashburton, New Zealand  <http://www.nzsock.co.nz/shop/cat-15/LifeSocks.htm> | Lifestyle sock -NZD $44.95  Base Med -  NZD $39.95  Airborne Quarter –  NZD $39.95  Airborne Lite -NZD $34.95  Protective Plus -  NZD$44.95 | **Lifestyle Med:**  55% Merino Wool  29% Nylon  9% Elastic  4% SeaCell active  3% Lycra  **BaseMed: Wormen & Men**  60% Merino Wool  21% Nylon  9% Elastic  7% SeaCell active  3% Lycra  **AirborneMed Quarter**:  58% Merino Wool  25% Nylon  9% Elastic  5% SeaCell active  3% Lycra  **Protective Med:**  60% Merino Wool  30% Nylon  10% Elastic | Created for the medical market and developed in consultation with expert clinical advisors.  Produced with advanced, cutting-edge technology, and incorporating a unique combination of natural fibres. Socks deliver outstanding preventative & protective features.  • reduce friction, shear and pressure  • provide effective temperature & moisture management  • offer resistance against compression  • assist in providing a bacteriostatic environment  • adapt to the individual shape of the foot  • offer safe, warm, non-constrictive hosiery  • provide an important protective interface between the skin and the shoe  **Calf**: Non-restrictive double cuff for easy slide-on, minimal compression & maximum stretch  **Ankle**: Anatomical cushioning strategically placed throughout for added protection and comfort flex panel for ergonomic shaping  **Heel**: Y heel for an improved, wrinkle-free fit and pressure point reduction  **Instep**: Unique* double – density cushion padding to protect Dorsal bony prominences from shoe pressure. Target system highlights Dorsalis Pedis Pulse  **Sole**: Ribs and mesh panels to aid airflow and to regulate moisture & temperature. Mesh panels for increased flexibility  **Toe**: Seamless toe closure for ridge-free comfort. Positioning points for correct alignment of sock  Individual shaping for left and right foot  **Paladin therapeutic sock:** Medical socks have a special alignment points are knitted into the sock; these allow the correct location of the Dorsal protection pad, giving protection from shoe and lace pressure.  Machine technology allows sock to be shaped and place cushioning just where needed for foot protection and comfort.  Elastic Support System ensures:  • a form-fitting system  • minimal compression  • a snug, customised fit  • no bunching or ride-down  • reduce friction & shear forces  Uses Merino wool:  Merino promises  • a natural, healthy alternative to synthetic fibres  • superior softness & comfort  • warmth without weight  • odour resistance  • an ability to breathe  • temperature & moisture management  • strength & durability  • extra resilience for impact protection  Incorporates Seacell technology: Cellulose + Seaweed + Silver = SeaCell active  **Calf**: The double cuff, high-stretch top ensures minimal compression.  **Sole**: Mesh and ribs are knitted into the sole of the sock for moisture management and temperature control. | New Zealand Ministry of Health (2004). Diabetes Toolkit. Wellington:  Ministry of Health. New Zealand.  Bayley, A. (2006). Pharmac Seminar  Series; The ischaemic diabetic foot  presentation. Wellington, New Zealand.  Jirkovska A, Boucek P. et al. (2001). Identification of patients at risk for diabetic foot. A comparison of standardized noninvasive testing with routine practice at community diabetes clinics. Journal of Diabetes and its complications 15:63-68  Burns, P., Gough, S., Bradbury, A.W.  (2003) Management of Peripheral arterial disease in primary care. BMJ, 326 584(5)  American College Foot & Ankle Surgeons (2006). www.footphysicians.com/diabetes.  “Global Burden of foot disease and  amputation in diabetic patients.” (2006)  www.medicalnewstoday.com cited in www.who.int.diabetes/en  Pan American Health Organisation  (PAHO), Regional Office of the World  Health Organisation, PAHO Today,  Newsletter of the Pan American Health  Organization, (Nov 2005) www.paho.org/  English/DD/PIN/ptoday20_nov05.htm  Ragnarson, M., Tennvall, G., Apelqvist,  J. (2001) Prevention of diabetes-related  foot ulcers and amputations: a cost-utility analysis based on Markov model simulations. Diabetologia, 44: 2077-2087.  Royal College of General Practitioners  (2003). Clinical guideline for type 2  Diabetes; Prevention and Management  of Foot Problems. Clinical Guidelines and  Evidence Review. www.rcgp.org.uk  New Zealand Diabetes Guidelines;  NZ Ministry of Health (2003):  Management of type 2 Diabetes. Evidence based Best Practice Guideline. New Zealand Guidelines Group; p. 67-77  11. Scottish Intercollegiate Guidelines  Network (2001). Management of Diabetes. www.sign.ac.uk  Armstrong, D. G., Lavery, L.A., Harkless, L.B., & Van Houtum, W.H. (1997) Amputation and reamputation of the diabetic foot. Journal American Podiatric Medical Association, 87 (6), 255-259  Bayley A. (2006). Pharmac Seminar series; The ischaemic diabetic foot. Wellington, New Zealand. |
| SensiFoot | JOBST, USA  <http://www.jobst.com> | Sensifoot Knee length –  USD $11.47 - $12.67  Sensifoot crew – USD $10.26/$11.17  Sensifoot mini crew –  USD $9.45/$10.05 | Acrylic | "The Complete Diabetic Sock"  “Offers more than ‘regular socks”:   - Non-irritating, smooth toe seam - Extra padding in the foot, heel and toe - Moisture-wicking acrylic multi-fiber yarns - Antibacterial, antifungal finish - Non-constricting mild compression   SensiFoot makes proper foot care an easy & comfortable part of any daily diabetes routine.   - Flat, low profile toe seam reduces irritation and pressure on toes - Acrylic padding reduces friction and provides cushioning for comfort - Acrylic yarn wicks away moisture to help keep feet cool and dry - Antimicrobial finish helps prevent growth of bacteria and fungi on the sock and helps eliminate odour - Non-constricting, mild gradient compression helps prevent sliding and bunching which can cause pressure points against the skin - Non-binding top welt won’t restrict circulation | Website provides medical links to:  JOBST Vascular Center,  American Venous form  American college of Phlebology  National Lymphedema Network. |
| DIFOPREV® | LVM Technologies, Italy  [www.lvmtechnologies.com](http://www.lvmtechnologies.com) | Difoprev socks -  NZD $99.00  (UK £49.20 in 2012)  Refills –  NZD $79.00 | Sock made from special fabric & glycoprotein solution = to 150ml of emollient cream  Use of nanotechnology  8.5% Cotone  4.5% meryl®  1% acrylic  0.5% Polyester | Goal: the prevention & restoration of foot’s epidermis  Clinical test were made in hospitals and universities and they demonstrated:   - a drastic improvement of the keratosis - elasticity and integrity of the skin is  improved - best results in TEWL parameters - improved microcirculation - there are no collateral effects   Advantages:   - absolutely invisible - practical and easy, and cause no change in daily life - Results can be seen in a little time - Application can be ended without any negative effect - Great help for doctors in emphasizing to patients the need for good prevention - Use can be adapted to every patient | Dr Stephen R Young, (2008) Difoprev Pilot study Final Report  G Pirotta (2008) Skin Delivery of Active Ingredients from Fabrics Chapter 52 Skin Barrier: Chemistry of Delivery Systems pp 551 – 556  B. Elisa et al (2008) Use of Nanotechnology-Designed Footsock in the Management of Preulcerative Conditions in theDiabetic Foot: Results of a Single, Blind Randomized Study The International Journal of Lower Extremity Wounds Volume 7 Number 2 82-87  DH Richie (2008) Therapeutic Hosiery: An essential component of footwear for the pathologic foot Podiatry Management 123-33  A Piagggesi et al (2009) Measurements in Diabetic Foot Journal of Wound Technology 3 19 -24 |
| Thenar Gel Protection diabetic socks & Full gel protection diabetic socks  20 -30 mmHg diabetic support socks (JG-976)  Non-binding diabetic Crew Sock (JG-992 & JG-991) | Tsung Hau, Taiwan  [www.footnurse.com.tw](http://www.footnurse.com.tw) | Thenar Gel Protection diabetic socks – USD $5.94 | 86% Bamboo 12% Charcoal Nylon 2%  (Lycra spandex)。  46% Lyocell  25% Polyester  19% Nylon  10%  Spandex(Lycra)  80% Acrylic  10% Nylon  10% Spandex | Thenar Gel Insole Gel- disperse forefoot pressure, preventing formation of corns  Sole coated with gel – Provide protection from trauma and repair severe dry feet of diabetic  **Ankle**: Loose welt – prevent from constricting the foot with optimum comfort & help blood flow smoothly  **Sole**: Terry cushion – help reducing blisters & minimizing foot irritation  Material: Bamboo Charcoal yarn – restrain bacterial breeding & reduce odour.  Seamless construction. Graduated Compression – improve Blood Circulation  Lyocell Yarn – antistatic, Sweat absorbing  **Toe**: seamless protect toes  **Calf**: Loose Cuff Comfortable  1. Gradual compression of diabetic support socks can reduce swelling in legs, ankles and feet.  2. 20-30mmHg Diabetic support socks can encourage circulatory wellness.  3. Gradual compression help improve poor blood circulation keeping warm.  4. Non-irritating toe seam help reduce blisters and help minimize foot irritation.  5. Lycra spandex is used for durability, excellent stretch and great fit.  **Calf**: Non-binding cuff, Loose feature at the leg  **Heel & sole**: terry cushion padding  1. Comfortable top band technology prevents from constricting the foot with optimum comfort and helps blood flow smoothly.  2. Sole protection with thick cushioning in sole is for maximum protection against shocks, impacts and blisters caused by sudden stops and starts.  3. Breathability with cooling channels promotes air flow for cooler, direr feet.  4. Roomy feature at the leg for excessive edema condition.  5. Acrylic fiber helps spread moisture out across fabric.  6. White color feature helps notice the wound condition. |  |
| Truform Trusoft Diabetic sock  Truform Comfort sock  Truform Casual & Atheletic socks 15 – 20mmHg | Truform, USA  [www.truform.com](http://www.truform.com) | Trusoft Diabetic Mini Crew sock  USD $13.75  Trusoft Crew length 8-15– USD $15.95  Trusoft Calf length USD $18.15  Comfort Care Casual sock (#1914) –  USD $15.95  15 -20 mmHG Casual & Athletic Socks (#1933) –  USD $21.89 | 73% acrylic,  22% Nylon,  5% Spandex  100% cotton  60% acrylic,  33% Nylon,  7% Spandex | Surround feet with full cushioned comfort. Non-constricting, gentle, graduated compression helps keep sock in place to prevent bunching and sliding. Moisture wicking yarns, stay fresh treatment and low profile toe closure help protect and comfort sensitive feet – ideal for diabetic patient.  Antibacterial, anti-fungal finish helps reduce odour.  **Calf:**  Gentle graduated compression  **Toe:** low profile, non-irritating toe seam  **Heel & Sole:** Full-cushioned foot  Designed for anyone with special needs in foot care. Non-constricting, gentle, elasticity helps keep sock in place to prevent bunching and sliding. The natural cotton material helps reduce irritation and blistering and provides exceptional comfort, easy care and excellent absorbancy. Ideal for the diabetic patient, or anyone with sensitive feet issues. Excellent cover for use with medicinal or softening creams. Suitable for wear at night for warmth. Thick cotton construction provides comfort and protection.  **Calf:** Non-binding & comfortable. Cut extra wide & extra deep  **Toe:**  Seamless toe closure.  TRUFORM support socks are designed to help improve circulation and reduce leg discomfort. Full-cushioned foot, moisture wicking fibers and anti-bacterial, stay-fresh finish provide all-day wearing comfort. Excellent for business-casual, or leisure activities that require comfort and support.  **Calf:** Therapeutic graduated compression  Moderate support.  **Sole**: Comfortable full-cushioned foot |  |
| **Sock type/Name** | **Manufacturer** | **Cost** | **Material content** | **Manufacturer’s claims** | **Supporting Evidence Trials** |
| Dr Scholls’s Diabetes & circulatory socks | Dr Scholl,  USA  [www.drschollssocks.com](http://www.drschollssocks.com) | Diabetes & circulatory socks  USD $ 4.99 | 92% Polyester, 7% Nylon,  1% Lycra/spandex | A sock perfect for Diabetics, those suffering from circulatory issues, sensitive skin or anyone who prefers a soft, non-binding sock. Moisture-dispersion technology keeps feet dry and anti-microbial technology keeps socks fresh. Durable and easy to put on, these socks provide the foot health benefits you need every day  **Calf**: a soft, comfortable, non-binding ‘morpul’ top that conforms to your legs and won't restrict circulation.  **Sole**: cushioned on the bottom to protect sensitive feet.  **Toe**: Smooth toe seam to add comfort and reduce irritation  Dr Scholl’s diabetic socks have been given the American Podiatric Medical Association stamp of approval for being an exceptional sock to promote quality foot health |  |
| SmartKnit seamless socks | SmartKnits, USA  [www.smartknit.com](http://www.smartknit.com)  [www.diabeticsocks.com](http://www.diabeticsocks.com) | SmartKnit Seamless Diabetic Mini-Crew Socks – USD $12.95  AUD $27  SmartKnit Seamless Diabetic Crew Socks –  USD $13.45  AUD $29  SmartKnit Seamless Diabetic over the calf socks USD$15.95  AUD $34 | White CoolMax: 97% CoolMax Polyester 3% Spandex;  Black Navy & Grey X-Static: 97% X-Static polyester blend 3% Spandex. | Completely free of seams, these patented socks fit like a second skin and are recommended for people with diabetes, arthritis, or any condition where protection from irritation is important. Moisture-wicking fibers wick away moisture.  Lycra® blend.  **Calf**: Form-Fitting Design - fit like a second skin, with no wrinkling or bunching and help in reducing the chances of circulation restriction  Non-Binding Top - Elastic-free "halo top" helps prevent the painful leg indentations often caused by elastic bands that fit too tight  **Toe**: Seamless Toe - prevents blister-causing pressure points often caused by bulky and uncomfortable sock seams  X-STATIC silver fibers in coloured sock options - prevent odor in the sock and are thermodynamic (cooler in the summer and warmer in the winter) |  |
| TheraSock | Therafirm, USA  [www.therafirm.com](http://www.therafirm.com) | TheraSock Comfort System Plus Socks – USD $ 10.95  TheraSock Comfort System Lite Socks – USD $8.95  Therasock Care Sox Plus Socks – USD $9.45  TheraSock Double Sock System Socks –  Mini Crew –  USD $10.49  Crew - USD $12.95 | Hi-bulk acrylic/nylon/spandex | TheraFrim wrap legs in graduated compression, with most pressure at the ankle with pressure gradually decrease up the leg.  **TheraSock Comfort System Plus Socks**   - Dense, heavy, comfort padding - provides extra buffer zone of protection - High-tech fibers - wick away moisture and inhibit bacterial growth for a healthier sock environment - Seamless interior - ensures there are no ridges or pressure against the skin - Wears especially well with properly fitted extra depth diabetic shoes   **TheraSock Comfort System Lite Socks**   - Seams on the exterior of the sock, wearers enjoy a completely seam-free feel - no ridges or pressure on the skin. - Soft, light padding - extra comfort and relief without extra bulk - High-tech fibers - wick away moisture and inhibit bacterial growth for a healthier sock environment   **Therasock Care Sox Plus Socks**  Designed especially for people with over-sized feet and lower legs, stretching up to 23" in circumference and provide extra padding and pressure relief. Perfect for use over bandages, dressings & casts. Features:   - Stretchable and non-binding materials - totally non-binding for proper circulation - Extra comfort padding provides a buffer zone of protection - Flat toe seam - reduces irritation - High-tech fibers - wick away moisture and inhibit bacterial growth for a healthier sock environment   **TheraSock Double Sock System Mini-Crew Socks,** by WrightSock   - Double layer construction "sock within a sock design" - reduces friction against the feet, providing maximum protection for sensitive feet - White inner liner - protects the skin from dyes and additives - High-tech fibers - wick away moisture and inhibit bacterial growth for a healthier sock environment |  |
